# 5-Chloro­quinolin-8-yl furan-2-carboxyl­ate

**DOI:** 10.1107/S1600536813005667

**Published:** 2013-03-06

**Authors:** Rodolfo Moreno-Fuquen, Geraldine Hernandez, Javier Ellena, Carlos A. De Simone, Juan C. Tenorio

**Affiliations:** a Departamento de Química, Facultad de Ciencias, Universidad del Valle, Apartado 25360, Santiago de Cali, Colombia; b Instituto de Física de São Carlos, IFSC, Universidade de São Paulo, USP, São Carlos, SP, Brazil

## Abstract

In the title compound, C_14_H_8_ClNO_3_, the central ester CO_2_ group is twisted away from the quinoline and furoyl rings by 57.46 (5) and 2.0 (1)°, respectively. In the crystal, mol­ecules are linked by weak C—H⋯O inter­actions, forming chains in [001].

## Related literature

For medicinal, anti­fungal, anti­bacterial, anti­cancer and luminiscent properties of the quinoline ring, see: Somvanshi *et al.* (2008 ▶), Biavatti *et al.* (2002 ▶), Towers *et al.* (1981 ▶), Shen *et al.* (1999 ▶) and Montes *et al.* (2006 ▶), respectively. For similar structures, see: Lei (2006 ▶; 2007 ▶). For hydrogen-bonding notation, see: Etter (1990 ▶); Nardelli (1995 ▶).
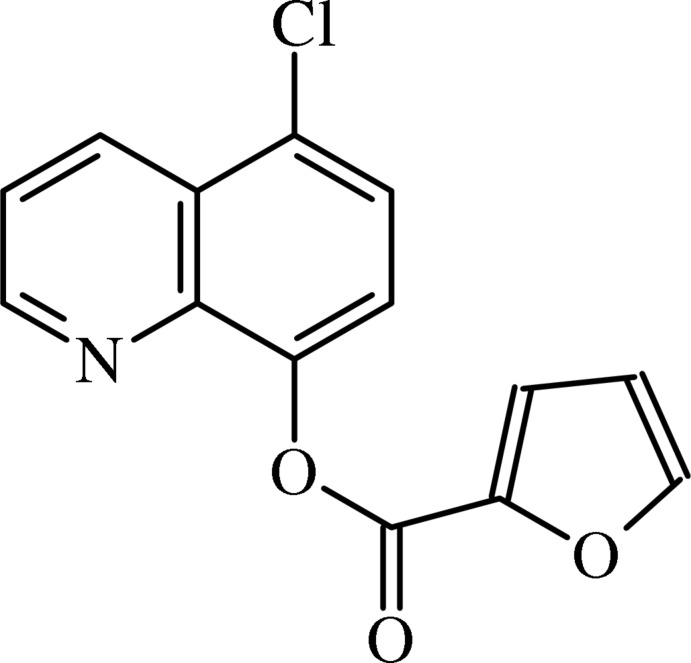


## Experimental

### 

#### Crystal data

C_14_H_8_ClNO_3_
*M* *_r_* = 273.66
Monoclinic, 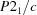

*a* = 4.0714 (1) Å
*b* = 23.7463 (7) Å
*c* = 12.7698 (4) Å
β = 102.113 (1)°
*V* = 1207.11 (6) Å^3^
*Z* = 4
Mo *K*α radiation
μ = 0.32 mm^−1^
*T* = 295 K
0.35 × 0.09 × 0.09 mm

#### Data collection

Nonius KappaCCD diffractometer
4385 measured reflections
2440 independent reflections
1906 reflections with *I* > 2σ(*I*)
*R*_int_ = 0.017

#### Refinement

*R*[*F* ^2^ > 2σ(*F* ^2^)] = 0.043
*wR*(*F* ^2^) = 0.119
*S* = 1.03
2440 reflections
172 parameters
H-atom parameters constrained
Δρ_max_ = 0.16 e Å^−3^
Δρ_min_ = −0.29 e Å^−3^

### 

Data collection: *COLLECT* (Nonius, 2000 ▶); cell refinement: *SCALEPACK* (Otwinowski & Minor, 1997 ▶); data reduction: *DENZO* (Otwinowski & Minor, 1997 ▶) and *SCALEPACK*; program(s) used to solve structure: *SHELXS97* (Sheldrick, 2008 ▶); program(s) used to refine structure: *SHELXL97* (Sheldrick, 2008 ▶); molecular graphics: *ORTEP-3 for Windows* (Farrugia, 2012 ▶) and *Mercury* (Macrae *et al.*, 2006 ▶); software used to prepare material for publication: *WinGX* (Farrugia, 2012 ▶).

## Supplementary Material

Crystal structure: contains datablock(s) I, global. DOI: 10.1107/S1600536813005667/gg2111sup1.cif

Structure factors: contains datablock(s) I. DOI: 10.1107/S1600536813005667/gg2111Isup2.hkl

Supplementary material file. DOI: 10.1107/S1600536813005667/gg2111Isup3.cml

Additional supplementary materials:  crystallographic information; 3D view; checkCIF report

## Figures and Tables

**Table 1:** Hydrogen-bond geometry (Å, °)

*D*—H⋯*A*	*D*—H	H⋯*A*	*D*⋯*A*	*D*—H⋯*A*
C14—H14⋯O2^i^	0.93	2.47	3.371 (2)	162

Symmetry code: (i) 
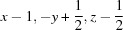
.

**Fig. 1.**
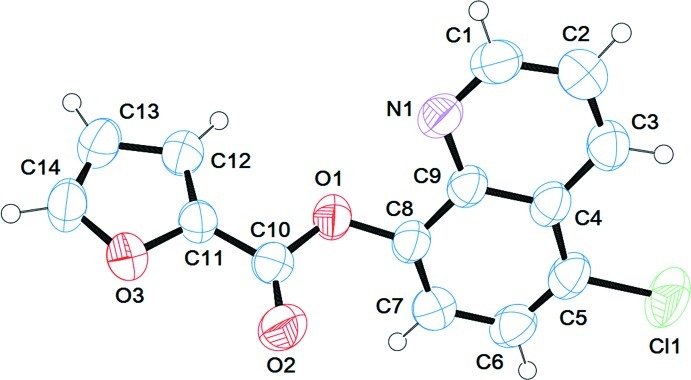
Molecular conformation and atom numbering scheme for the title compound (I) with displacement ellipsoids drawn at the 50% probability level. H atoms are shown as spheres of arbitrary radius.

**Fig. 2.**
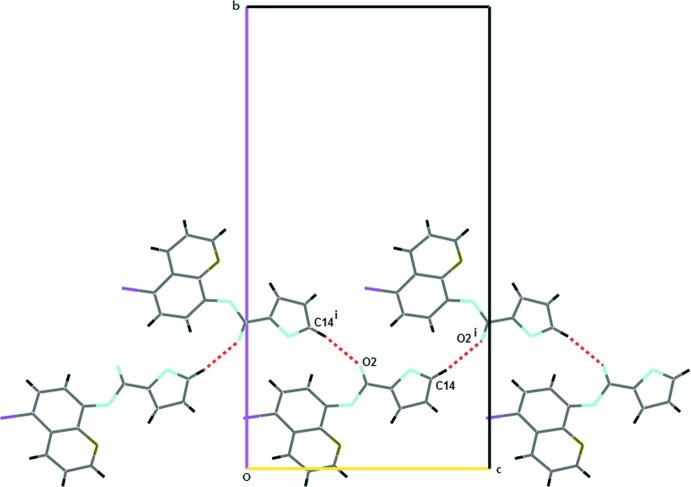
Part of the crystal structure of (I), showing the formation of helical chains which align along [001]. Symmetry code: (i) x-1,-y+1/2,+z-1/2.

## supplementary crystallographic information

### Comment

The title compound, C_14_H_8_ClNO_3_ [5-chloroquinolin-8-yl-furan-2-carboxylate] (I), is part of a series of studies on the structural properties of the quinoline fragment developed by our research group. The quinoline ring exhibits a key factor responsible for a wide range of medicinal (Somvanshi *et al.*, 2008), antifungal (Biavatti *et al.*, 2002) and antibacterial (Towers *et al.*, 1981) properties. The quinoline ring has been also used in anticancer studies (Shen *et al.*, 1999), as well as its derivatives have been exploited for their luminescent properties as organic light-emitting diodes (OLED) materials (Montes *et al.*, 2006). The molecular structure of (I) is shown in Fig. 1. Bond lengths and bond angles of (I) show marked similarity with other 8-Hydroxyquinoline benzoates reported in the literature such as 8-Quinolyl benzoate and 2-Methylquinolin-8-yl 2-nitrobenzoate (Lei, 2006 and 2007). The central ester moiety, C8/O1/C10/O2/C11, is essentially planar with a r.m.s deviation of fitted atoms of 0.007 Å. The ester group is twisted away from the quinoline and furoyl rings by 57.45 (5)° and 2.0 (1)°, respectively. The crystal packing shows no classical hydrogen bonds. The crystal packing is stabilized by weak C-H···O intermolecular interactions, forming C(6) chains along [001] (see Fig. 2; Etter, 1990). The C14 atom of the furoyl ring at (x,y,z) acts as a hydrogen-bond donor to carbonyl atom O2 at (x-1,-y+1/2,+z-1/2) (see Table 1; Nardelli, 1995).

### Experimental

The reagents and solvents for the synthesis were obtained from the Aldrich Chemical Co. and were used without additional purification. In a 100 ml round bottom flask 2-furoyl chloride (1.564 mmol, 0.204 g) and 5-chloro-8hidroxyquinoline (1.564 mmol, 0.260 g) in equimolar amounts were mixed. The mixture was left to reflux in 20 ml of acetonitrile in constant stirring for about two hours, adding small amounts of pyridine as catalyst. A colourless solid was obtained after leaving the solvent to evaporate. IR spectra were recorded on a FT—IR SHIMADZU IR-Affinity-1 spectrophotometer. Colourless crystals; m.p 389 (1) K. IR (KBr) 3127 cm^-1^, 3097 cm^-1^ (aromatic C—H); 1743 cm^-1^ (ester C=O), 1299 cm^-1^ (ester C-O); 1588 cm^-1^, 1495 cm^-1^, 1392 cm^-1^ (amine C—N); 1467 cm^-1^ (furan C-O); 1181 cm^-1^ (C=C); 936 cm^-1^ (Cl-C).

### Refinement

All the H-atoms attached to C atoms were positioned at geometrically idealized positions and treated as riding with C—H= 0.93 Å and *U*_iso_(H) = 1.2 *U*_eq_(C).

### Crystal data

**Table:**

C_14_H_8_ClNO_3_	*F*(000) = 560
*M**_r_* = 273.66	*D*_x_ = 1.506 Mg m^−^^3^
Monoclinic, *P*2_1_/*c*	Melting point: 389(1) K
Hall symbol: -P 2ybc	Mo *K*α radiation, λ = 0.71073 Å
*a* = 4.0714 (1) Å	Cell parameters from 2589 reflections
*b* = 23.7463 (7) Å	θ = 3.1–26.3°
*c* = 12.7698 (4) Å	µ = 0.32 mm^−^^1^
β = 102.113 (1)°	*T* = 295 K
*V* = 1207.11 (6) Å^3^	Needle, colourless
*Z* = 4	0.35 × 0.09 × 0.09 mm

### Data collection

**Table:**

Nonius KappaCCD diffractometer	1906 reflections with *I* > 2σ(*I*)
Radiation source: fine-focus sealed tube	*R*_int_ = 0.017
Graphite monochromator	θ_max_ = 26.3°, θ_min_ = 3.1°
CCD rotation images, thick slices scans	*h* = −5→5
4385 measured reflections	*k* = −29→27
2440 independent reflections	*l* = −15→15

### Refinement

**Table:**

Refinement on *F*^2^	Primary atom site location: structure-invariant direct methods
Least-squares matrix: full	Secondary atom site location: difference Fourier map
*R*[*F*^2^ > 2σ(*F*^2^)] = 0.043	Hydrogen site location: inferred from neighbouring sites
*wR*(*F*^2^) = 0.119	H-atom parameters constrained
*S* = 1.03	*w* = 1/[σ^2^(*F*_o_^2^) + (0.0636*P*)^2^ + 0.2631*P*] where *P* = (*F*_o_^2^ + 2*F*_c_^2^)/3
2440 reflections	(Δ/σ)_max_ < 0.001
172 parameters	Δρ_max_ = 0.16 e Å^−^^3^
0 restraints	Δρ_min_ = −0.29 e Å^−^^3^

### Special details

**Table:**

Geometry. All esds (except the esd in the dihedral angle between two l.s. planes) are estimated using the full covariance matrix. The cell esds are taken into account individually in the estimation of esds in distances, angles and torsion angles; correlations between esds in cell parameters are only used when they are defined by crystal symmetry. An approximate (isotropic) treatment of cell esds is used for estimating esds involving l.s. planes.
Refinement. Refinement of *F*^2^ against ALL reflections. The weighted *R*-factor *wR* and goodness of fit *S* are based on *F*^2^, conventional *R*-factors *R* are based on *F*, with *F* set to zero for negative *F*^2^. The threshold expression of *F*^2^ > σ(*F*^2^) is used only for calculating *R*-factors(gt) *etc*. and is not relevant to the choice of reflections for refinement. *R*-factors based on *F*^2^ are statistically about twice as large as those based on *F*, and *R*- factors based on ALL data will be even larger.

### Fractional atomic coordinates and isotropic or equivalent isotropic displacement parameters (Å^2^)

**Table:**

	*x*	*y*	*z*	*U*_iso_*/*U*_eq_	
Cl1	0.86765 (17)	0.10906 (3)	1.01429 (4)	0.0852 (3)	
O3	−0.0985 (4)	0.21415 (5)	0.32844 (10)	0.0617 (4)	
O2	0.3216 (4)	0.22368 (6)	0.53277 (11)	0.0735 (4)	
O1	0.1366 (3)	0.13797 (5)	0.56894 (10)	0.0590 (4)	
N1	0.5632 (4)	0.05097 (6)	0.61584 (11)	0.0518 (4)	
C9	0.5359 (4)	0.08729 (7)	0.69653 (13)	0.0442 (4)	
C3	0.9075 (5)	0.02976 (8)	0.82623 (15)	0.0578 (5)	
H3	1.0231	0.0221	0.8956	0.069*	
C4	0.7073 (4)	0.07857 (7)	0.80464 (13)	0.0470 (4)	
C10	0.1468 (4)	0.18356 (7)	0.50701 (13)	0.0471 (4)	
C8	0.3285 (5)	0.13513 (7)	0.67304 (14)	0.0491 (4)	
C6	0.4679 (5)	0.16529 (8)	0.85564 (15)	0.0592 (5)	
H6	0.4485	0.1917	0.9078	0.071*	
C2	0.9304 (5)	−0.00590 (9)	0.74516 (17)	0.0630 (5)	
H2	1.0611	−0.0383	0.7585	0.076*	
C12	−0.2882 (5)	0.13064 (8)	0.36831 (15)	0.0540 (4)	
H12	−0.3217	0.0982	0.4054	0.065*	
C11	−0.0828 (4)	0.17374 (7)	0.40520 (13)	0.0456 (4)	
C7	0.2944 (5)	0.17315 (8)	0.74910 (16)	0.0568 (5)	
H7	0.1566	0.2044	0.7310	0.068*	
C14	−0.3227 (6)	0.19455 (10)	0.24173 (16)	0.0668 (6)	
H14	−0.3837	0.2136	0.1769	0.080*	
C5	0.6638 (5)	0.11902 (8)	0.88183 (14)	0.0540 (5)	
C1	0.7556 (5)	0.00645 (8)	0.64139 (16)	0.0592 (5)	
H1	0.7767	−0.0184	0.5869	0.071*	
C13	−0.4431 (5)	0.14454 (10)	0.26179 (16)	0.0654 (5)	
H13	−0.5989	0.1229	0.2149	0.078*	

### Atomic displacement parameters (Å^2^)

**Table:**

	*U*^11^	*U*^22^	*U*^33^	*U*^12^	*U*^13^	*U*^23^
Cl1	0.1006 (5)	0.1148 (5)	0.0337 (3)	0.0015 (4)	−0.0007 (3)	−0.0047 (3)
O3	0.0868 (9)	0.0501 (7)	0.0449 (7)	0.0020 (6)	0.0064 (7)	0.0084 (5)
O2	0.1084 (11)	0.0582 (8)	0.0486 (8)	−0.0317 (8)	0.0048 (7)	0.0004 (6)
O1	0.0734 (8)	0.0500 (7)	0.0447 (7)	−0.0140 (6)	−0.0080 (6)	0.0094 (6)
N1	0.0647 (9)	0.0509 (8)	0.0395 (8)	−0.0130 (7)	0.0102 (7)	−0.0050 (6)
C9	0.0526 (10)	0.0451 (9)	0.0344 (8)	−0.0115 (7)	0.0079 (7)	0.0001 (7)
C3	0.0614 (11)	0.0620 (11)	0.0475 (10)	−0.0011 (9)	0.0056 (8)	0.0094 (9)
C4	0.0513 (10)	0.0520 (10)	0.0369 (8)	−0.0089 (8)	0.0077 (7)	0.0027 (7)
C10	0.0581 (10)	0.0434 (9)	0.0403 (9)	−0.0011 (8)	0.0115 (7)	0.0006 (7)
C8	0.0585 (10)	0.0470 (9)	0.0383 (9)	−0.0095 (8)	0.0023 (7)	0.0038 (7)
C6	0.0748 (13)	0.0603 (11)	0.0448 (10)	−0.0083 (10)	0.0175 (9)	−0.0107 (8)
C2	0.0687 (12)	0.0553 (11)	0.0659 (13)	0.0038 (9)	0.0160 (10)	0.0043 (9)
C12	0.0557 (10)	0.0539 (10)	0.0499 (10)	−0.0021 (8)	0.0051 (8)	0.0021 (8)
C11	0.0543 (10)	0.0435 (9)	0.0395 (9)	0.0061 (7)	0.0109 (7)	0.0039 (7)
C7	0.0650 (11)	0.0503 (10)	0.0543 (11)	−0.0005 (9)	0.0108 (9)	0.0003 (8)
C14	0.0805 (14)	0.0729 (14)	0.0418 (10)	0.0171 (11)	0.0008 (9)	0.0058 (9)
C5	0.0609 (11)	0.0663 (12)	0.0339 (9)	−0.0085 (9)	0.0080 (8)	−0.0027 (8)
C1	0.0717 (12)	0.0515 (10)	0.0564 (12)	−0.0082 (9)	0.0181 (9)	−0.0094 (9)
C13	0.0626 (12)	0.0755 (14)	0.0508 (11)	0.0014 (10)	−0.0047 (9)	−0.0035 (10)

### Geometric parameters (Å, º)

**Table:**

Cl1—C5	1.7370 (18)	C4—C9	1.425 (2)
O1—C8	1.395 (2)	C5—C6	1.358 (3)
O1—C10	1.347 (2)	C6—C7	1.408 (3)
O2—C10	1.193 (2)	C6—H6	0.9300
O3—C11	1.364 (2)	C7—C8	1.355 (3)
O3—C14	1.361 (2)	C7—H7	0.9300
N1—C1	1.316 (2)	C8—C9	1.410 (2)
N1—C9	1.366 (2)	C10—C11	1.452 (2)
C1—C2	1.397 (3)	C11—C12	1.343 (2)
C1—H1	0.9300	C12—C13	1.413 (3)
C2—C3	1.356 (3)	C12—H12	0.9300
C2—H2	0.9300	C13—C14	1.330 (3)
C3—C4	1.410 (3)	C13—H13	0.9300
C3—H3	0.9300	C14—H14	0.9300
C4—C5	1.414 (3)		
			
C14—O3—C11	105.45 (15)	C1—C2—H2	120.3
C10—O1—C8	121.21 (13)	C11—C12—C13	106.20 (17)
C1—N1—C9	117.31 (15)	C11—C12—H12	126.9
N1—C9—C8	119.22 (14)	C13—C12—H12	126.9
N1—C9—C4	122.55 (16)	C12—C11—O3	110.63 (15)
C8—C9—C4	118.23 (15)	C12—C11—C10	132.30 (16)
C2—C3—C4	119.58 (17)	O3—C11—C10	117.05 (15)
C2—C3—H3	120.2	C8—C7—C6	119.90 (18)
C4—C3—H3	120.2	C8—C7—H7	120.0
C3—C4—C5	125.02 (16)	C6—C7—H7	120.0
C3—C4—C9	117.01 (16)	C13—C14—O3	111.11 (17)
C5—C4—C9	117.96 (16)	C13—C14—H14	124.4
O2—C10—O1	124.77 (16)	O3—C14—H14	124.4
O2—C10—C11	127.56 (16)	C6—C5—C4	122.09 (17)
O1—C10—C11	107.65 (14)	C6—C5—Cl1	119.07 (15)
C7—C8—O1	122.00 (17)	C4—C5—Cl1	118.83 (15)
C7—C8—C9	122.11 (16)	N1—C1—C2	124.20 (18)
O1—C8—C9	115.62 (15)	N1—C1—H1	117.9
C5—C6—C7	119.68 (17)	C2—C1—H1	117.9
C5—C6—H6	120.2	C14—C13—C12	106.60 (18)
C7—C6—H6	120.2	C14—C13—H13	126.7
C3—C2—C1	119.34 (19)	C12—C13—H13	126.7
C3—C2—H2	120.3		
			
C1—N1—C9—C8	−179.10 (16)	C14—O3—C11—C10	178.79 (16)
C1—N1—C9—C4	0.3 (2)	O2—C10—C11—C12	179.9 (2)
C2—C3—C4—C5	−179.79 (18)	O1—C10—C11—C12	1.4 (3)
C2—C3—C4—C9	0.4 (3)	O2—C10—C11—O3	1.4 (3)
N1—C9—C4—C3	−0.7 (2)	O1—C10—C11—O3	−177.05 (15)
C8—C9—C4—C3	178.71 (15)	O1—C8—C7—C6	173.41 (16)
N1—C9—C4—C5	179.51 (16)	C9—C8—C7—C6	−0.4 (3)
C8—C9—C4—C5	−1.1 (2)	C5—C6—C7—C8	−1.2 (3)
C8—O1—C10—O2	2.3 (3)	C11—O3—C14—C13	−0.1 (2)
C8—O1—C10—C11	−179.13 (15)	C7—C6—C5—C4	1.6 (3)
C10—O1—C8—C7	59.4 (2)	C7—C6—C5—Cl1	−178.39 (14)
C10—O1—C8—C9	−126.42 (17)	C3—C4—C5—C6	179.78 (18)
N1—C9—C8—C7	−179.07 (17)	C9—C4—C5—C6	−0.4 (3)
C4—C9—C8—C7	1.5 (3)	C3—C4—C5—Cl1	−0.2 (3)
N1—C9—C8—O1	6.8 (2)	C9—C4—C5—Cl1	179.55 (12)
C4—C9—C8—O1	−172.65 (14)	C9—N1—C1—C2	0.4 (3)
C4—C3—C2—C1	0.2 (3)	C3—C2—C1—N1	−0.6 (3)
C13—C12—C11—O3	0.0 (2)	O3—C14—C13—C12	0.1 (2)
C13—C12—C11—C10	−178.50 (18)	C11—C12—C13—C14	−0.1 (2)
C14—O3—C11—C12	0.0 (2)		

### Hydrogen-bond geometry (Å, º)

**Table:**

*D*—H···*A*	*D*—H	H···*A*	*D*···*A*	*D*—H···*A*
C14—H14···O2^i^	0.93	2.47	3.371 (2)	162

Symmetry code: (i) *x*−1, −*y*+1/2, *z*−1/2.
